# Stress, Health Behaviors, and Blood Pressure in College Students

**DOI:** 10.1002/hsr2.72243

**Published:** 2026-04-05

**Authors:** Ayman Tailakh, Ali Ahmad Ammouri

**Affiliations:** ^1^ School of Nursing California State University Los Angeles California USA; ^2^ Department of Adult Health Nursing, Faculty of Nursing The Hashemite University Zarqa Jordan

**Keywords:** college students, health behaviors, sleep, stress, United States of America

## Abstract

**Background:**

College students face multiple stressors, yet few studies have simultaneously examined associations between blood pressure, health behaviors (diet, physical activity, weight management, alcohol, tobacco, sleep), depressive symptoms, and perceived stress after multivariable adjustment. This cross‐sectional study investigated these associations in a diverse university sample.

**Methods:**

A convenience sample of 520 students from a public university in Southern California completed validated self‐report measures, including the Perceived Stress Scale (PSS‐10), Patient Health Questionnaire (PHQ‐9), and Hypertension Self‐Care Activity Level Effects (H‐SCALE), and underwent objective blood pressure, height, and weight measurements. Approximately 750 students were approached; 520 participated (69.3% participation rate). Multiple linear regression (*α* = 0.05) was used to identify predictors of stress while controlling for demographics, health behaviors, and clinical variables.

**Results:**

Participants (mean age 22.99 ± 4.78 years; 64.8% female; 53.5% Hispanic/Latino) had a mean stress score of 18.80 ± 6.19. In the multivariable model (F[21,225] = 7.426, *p* < 0.001, *R* ^2^ = 0.409; *n* = 247 complete cases), higher stress was significantly associated with higher diastolic blood pressure (*β* = 0.153, *p* = 0.030) and depressive symptoms (*β* = 0.509, *p* < 0.001), whereas adherence to a healthy diet (*β* = −0.118, *p* = 0.040) and weight management behaviors (*β* = −0.156, *p* = 0.008) was associated with lower stress. Systolic blood pressure, sleep duration, physical activity, smoking, and alcohol use were not significant predictors.

**Conclusions:**

In this university sample, elevated depressive symptoms and diastolic blood pressure were associated with higher perceived stress, whereas healthy dietary practices and weight management behaviors were associated with lower stress. These associations suggest potential targets for campus wellness programs; however, longitudinal studies are needed to establish temporal relationships and intervention efficacy.

According to the 2022 Stress in America survey, 48% of young adults aged 18–34 reported experiencing daily stress so severe that it interfered with their ability to function, a rate that increased to 54% by 2025 [1, 2]. Among college students, stress was the third most common concern for seeking counseling after anxiety and depression, respectively [3, 4]. College life poses numerous stressors that students regularly encounter, such as academic stress [5] and finances [6]. Many individuals use unhealthy coping strategies such as comfort eating, poor diet choices, smoking, binge drinking, and physical inactivity to manage stress. Binge drinking, defined as consuming five or more drinks for men or four or more drinks for women in about 2 h (Substance Abuse and Mental Health Services Administration [SAMHSA], 2022), is common among college students. Individuals experiencing stress are more likely to develop hypertension, cardiovascular disease, obesity, anxiety, and depression [7].

Despite recognition of these associations, limited research has simultaneously examined multiple modifiable health behaviors, objectively measured blood pressure, and depressive symptoms as correlates of perceived stress after multivariable adjustment. Guided by Lazarus and Folkman's [8] stress appraisal framework and McEwen's [9] allostatic load model, chronic stress is understood to trigger both maladaptive behavioral coping responses and cumulative physiological dysregulation across cardiovascular and neuroendocrine systems. Within this framework, health behaviors such as diet, physical activity, weight management, sleep, and substance use represent appraisal‐driven coping responses, while elevated blood pressure and depressive symptoms reflect downstream allostatic burden. This cross‐sectional study examined whether perceived stress was associated with depressive symptoms, blood pressure indices, sleep duration, and specific self‐care behaviors (H‐SCALE subscales) in a large, ethnically diverse university sample. We hypothesized that (1) higher depressive symptoms and (2) less healthy self‐care behaviors would be independently associated with higher perceived stress, and (3) elevated blood pressure.

Extensive research indicates that unhealthy behaviors such as e‐cigarette use [10], skipping meals [11], alcohol drinking, and binge drinking [7] are common among college students. Moreover, these behaviors could be associated with elevated stress levels and subsequent development of chronic health diseases such as hypertension, cardiovascular disease (CVD), diabetes, and obesity [12, 13]. In addition to unhealthy lifestyle choices, stress has been highly correlated with depression and anxiety [14] among college students.

Sleep is often curtailed during college. According to the American College Health Association [10], 42% of students reported averaging less than 7 h of sleep over the last 2 weeks. Additionally, one in four college students reported that sleep challenges affected their academic achievements. Prior research found that 70% of college students reported sleep deprivation, characterized by insufficient sleep duration or quality to support adequate alertness, often resulting in cognitive impairment and fatigue [15]. For young adults, sleep deprivation, typically defined as getting less than 7 h per night, is linked with poorer cognitive performance, mood disturbances, and impaired academic functioning (APA 2021 [3]). Furthermore, inadequate sleep has been linked to lower grade‐point averages, an increased risk of academic failure, and worsened mood [15].

Physical inactivity and poor diet are common in this population and may compound stress‐related outcomes. Only 38% of adults aged 18–24 meet recommended levels of physical activity [16], and nearly 20% were obese [17]. Additionally, 51.5% reported alcohol use and 44.4% engaged in binge drinking within the past month [18]. Tobacco use among young adults, especially e‐cigarettes, remains widespread, with 17.6% of those aged 18–24 reporting tobacco use [19]. Smoking is also more prevalent among college students who report higher levels of perceived stress [20].

Early adaptation of healthy lifestyles in young adulthood creates sustainable, healthy behavior patterns throughout life [21]. College students should be targeted for health prevention and promotion measures. In addition to adolescent's and young adults' lack of awareness of their current health status [22], lifestyle choices demonstrated by college students, particularly physical inactivity; high fat and sodium dietary intake; smoking; and alcohol drinking are known to contribute to an increased risk of subsequent development of stress‐related chronic health diseases, including CVD and hypertension, which are modifiable [23].

Given the significant morbidity and mortality associated with CVD, it is essential to examine lifestyle behaviors and stress levels on college campuses. Previous research has shown a positive correlation between lack of sleep [24] and depression [14] and increased stress among college students. However, few studies have simultaneously explored multiple modifiable lifestyle behaviors and their combined associations with stress, particularly in diverse student populations.

This study addresses this gap by specifically identifying which modifiable behaviors, such as adequate sleep, healthy diet, physical activity, alcohol and tobacco use, and weight management, are most strongly associated with perceived stress. Furthermore, the inclusion of a large, ethnically diverse sample from a public university enhances the generalizability and novelty of the study findings.

## 1 Methods

### 1.1 Design and Sample

A cross‐sectional, descriptive design was used for this study. A convenience sample of 520 college students was recruited at a comprehensive public university in Southern California. Participants were invited to the study through word of mouth, campus‐wide emails, fliers, social media posts, and posters throughout campus. Approximately 750 students were approached across these recruitment venues; 520 agreed to participate, yielding a 69.3% participation rate. The inclusion criteria were: (1) 18 years old or older, and (2) currently enrolled students. The exclusion criteria were: (1) having a physical or psychological impairment, (2) history of kidney disease, (3) history of secondary hypertension, and (4) currently pregnant. Students were recruited over a 3‐day period (Tuesday, Wednesday, Thursday) between 9:00 a.m. and 6:00 p.m. outside the Student Health Center.

The sample size was estimated based on the number of identified study variables and regression analysis. It was estimated that a minimum of 214 participants would be necessary to achieve a power of 0.80, a medium effect size (*ƒ* ² = 0.25), and an alpha of 0.05, when a regression analysis with 16 independent variables is used to predict the dependent variable (stress) [25]. The final regression model included 21 degrees of freedom due to the categorical predictors (race/ethnicity, marital status), which were expanded into multiple dummy variables when entered as model covariates. With *n* = 247 complete cases after listwise deletion, statistical power remained adequate ( > 0.80) for the observed effect size (*R*² = 0.409).

### 1.2 Data Collection

The University Institutional Review Board approved the study protocol (Application #14‐117; approved May 20, 2015). All participants were provided with informed consent before participation. The data collection was conducted during the Spring 2016 semester. The participants completed a series of questionnaires (with an average completion time of 20–30 min). These surveys included demographic information, brief health history, physical activity, perceived stress level, depression symptoms, health status, sleep hours, and diet. Clinical health status measures were obtained after questionnaire completion. Participants received a $5 gift card incentive for completing the measurements and the questionnaire.

### 1.3 Instruments

The demographic data included age, gender, marital status, employment, income, year in school, self‐reported average grade point average, and household size. Sleep duration was assessed using a single self‐reported item: based on responses to the question, “In the past 7 days, on average, how many hours do you sleep in a given 24‐h period?” While practical, this measure only captures sleep duration and does not account for sleep quality or disturbances, factors that could provide deeper insight into the relationship between sleep and perceived stress. Self‐rated health was also assessed based on one question. Students were asked to check mark their perceived general health as excellent, very good, good, fair, or poor.

The Perceived Stress Scale (PSS) was used to measure perceived stress level within the past month. This 10‐item questionnaire (PSS‐10) utilized a five‐point scale, with responses ranging from 0 (never) to 4 (very often). Responses were summed, and scores ranged from 0 to 40. Higher scores indicated greater perceived stress. Research has shown that the PSS is valid and reliable [26, 27]. Additionally, the PSS‐10 has been validated across diverse college student samples, demonstrating strong factorial and convergent validity [28, 29]. Cronbach's alpha reliability for the PSS‐10 in the current study was 0.76.

The level of depression was measured using the Patient Health Questionnaire (PHQ‐9). The PHQ‐9 is a nine‐item, self‐administered questionnaire that can be utilized for screening, diagnosis, and measuring the severity of depression [30]. The first nine items pertained to the question, “Over the last 2 weeks, how often have you been bothered by any of the following problems?” Responses were scored on a four‐point scale from 0 (not at all) to 3 (nearly every day). The score is the sum of the nine items, with a possible range of 0–27. A cut‐off PHQ‐9 score ≥ 10 is most commonly used for depression screening [31]. The PHQ‐9 has proven to be a valid instrument for diagnosing and assessing the severity of depression [32]. Moreover, it has demonstrated robust psychometric properties, including strong factorial and convergent validity, across various college student populations [33, 34]. In this study, Cronbach's alpha of PHQ‐9 was 0.91.

Lifestyle behaviors (i.e., physical activity, weight management, healthy diet, limiting alcohol intake, and tobacco use) were assessed using the validated Hypertension Self‐Care Activity Level Effects (H‐SCALE) [35, 36]. Although developed for hypertension self‐care, these behavioral domains (diet, activity, weight management, alcohol, and tobacco exposure) are also clinically meaningful for cardiometabolic risk reduction in young adults; therefore, we used the relevant subscales to characterize modifiable behaviors in this student sample. The H‐SCALE contains 31 items across six subscales designed to evaluate adherence to behaviors associated with lowering blood pressure risk. For this study, we specifically used five subscales: weight management, healthy diet, physical activity, alcohol intake, and tobacco exposure.

Weight management was assessed using a 10‐item subscale measuring behaviors such as reducing portion sizes, making healthier food substitutions, and exercising explicitly for weight loss over the past 30 days [35]. Response options range from strongly disagree (1) to strongly agree (5). Scores were summed (range 10–50); scores ≥ 40 indicate adherence to weight management.

Physical activity was assessed with two items. (1) “On how many of the past 7 days did you do at least 30 min of total physical activity?” and (2) “On how many of the past 7 days did you do a specific exercise activity (such as swimming, walking, or biking) other than routine activities around the house or as part of your work?” Responses for each item range from 0 to 7 days. The total score can range from 0 to 14. Participants who score eight or higher are adherent to physical activity.

Tobacco exposure was assessed with a two‐item H‑SCALE subscale, “How many of the past 7 days did you smoke a cigarette or cigar, even just one puff?” and to assess exposure to passive smoking, “How many of the past 7 days did you stay in a room or ride in an enclosed vehicle while someone was smoking?” Responses are summed (range 0–14); participants who score zero are adherent to tobacco exposure and no smoking. It is important to note that this measure does not distinguish between traditional cigarette/cigar smoking and the use of electronic cigarettes (vaping), a limitation that affects the interpretation of findings related to tobacco use.

Alcohol intake was assessed using a 3‐item H‑SCALE subscale that measured the (a) average number of days alcohol is consumed, (b) amount of alcohol consumed when drinking, and (c) maximum number of drinks consumed in 24 h in the last month. Respondents who score zero on all items are considered adherent/abstainers.

A healthy diet was measured using a 12‐item subscale reflecting adherence to dietary practices aligned with the Dietary Approaches to Stop Hypertension (DASH) guidelines, specifically low‐fat and low‐sodium consumption. An example item from the subscale is: “On how many of the past 7 days did you eat salty snacks (such as chips, salted nuts, or salted crackers)?” Responses ranged from 0 to 7 days. Of these items, nine were negatively worded and thus required reverse coding. After reverse coding, item responses were summed (possible total: 0–84) and converted to a mean score (range: 0–7), with higher scores indicating healthier dietary adherence. Participants with mean scores of 6 or higher were categorized as adherent (healthy diet), while those scoring below 6 were categorized as non‐adherent (unhealthy diet). In this study, Cronbach's alpha coefficient for maintaining ideal body weight, eating low‐salt diet, engaging in regular physical activity, limiting alcohol intake, and reducing tobacco use and exposure subscales were 0.91, 0.83, 0.84, 0.85, and 0.76, respectively.

After completing the questionnaire, the researcher measured each participant's BP, weight, and height. Blood pressure was measured using the Omron HEM‐705CP automatic BP monitor (HEM‐705CP, Omron Corporation, Tokyo, Japan). Participants were seated for 5 min with their backs supported and their feet flat on the floor. Blood pressure measurements were obtained after participants avoided caffeine, vigorous exercise, and smoking for at least 30 min when feasible. Appropriate cuff size was selected based on arm circumference. Three BP measurements were obtained on the left arm positioned at heart level, with 60‐second intervals between readings. The mean of the three readings was used for analysis. Hypertension was defined as a systolic blood pressure (SBP) greater than 140 mmHg, a diastolic blood pressure (DBP) greater than 90 mmHg, and/or a self‐reported diagnosis of or current treatment for hypertension.

Body mass index (BMI) was employed in the current study to assess weight status. BMI measures body fat based on height and weight calculation; that is, weight in kilograms divided by the square of height in meters (weight/height² (kg/m²)) [37]. Weight was measured to the nearest 0.2‐pound increments using the Omron HB‐40 Scale. Obesity is defined as a BMI of ≥ 30 kg/m², overweight as a BMI of 25–29.9 kg/m², normal weight as 18.5–24.9 kg/m², and underweight as < 18.5 kg/m² [37]. To ensure the reliability of weight measurements, scales were calibrated before each weight measurement to reduce reader error. Height was measured with a stadiometer without footwear. This device is designed to assess height to the nearest 0.5 cm.

### 1.4 Data Analysis

Data were analyzed using IBM SPSS Statistics (Version 22, IBM Corp., Armonk, NY). Descriptive statistics, including frequencies and means (± SD), were calculated to summarize demographic characteristics and key study variables. Pearson product‐moment correlations examined associations between continuous variables. *χ* ^2^ tests evaluated differences between categorical variables by gender, while independent‐samples *t*‐tests compared continuous variables by gender. For Table 2 analyses, categories were collapsed when needed, and Fisher's exact tests were used for small expected cell counts to avoid ad hoc subgroup exclusion. A multiple linear regression analysis was conducted to identify significant predictors of stress (PSS‐10), including gender, age, race/ethnicity, marital status, BMI, depression (PHQ‐9 scores), sleep duration, systolic blood pressure (SBP), diastolic blood pressure (DBP), self‐rated health, physical activity, healthy diet, weight management, alcohol intake, and cigarette smoking. Distributional assumptions were examined using histograms, skewness/kurtosis, and Q–Q plots. PSS‐10 scores approximated normality, and regression residuals showed no serious departures from normality. All statistical tests were two‐sided, and statistical significance was set at *α* = 0.05. Multicollinearity diagnostics indicated no serious violations (VIF < 2.0).

Missing data were addressed using listwise deletion. Of the 520 participants who completed study procedures, the final multiple linear regression model included 247 participants with complete data on all analysis variables, resulting in the exclusion of 273 cases (52.5%). Across individual variables, missingness ranged from 11.1% (e.g., race/ethnicity) to 32.2% (e.g., BMI), with biometric variables (e.g., BMI, blood pressure) contributing disproportionately. Listwise deletion was retained for the primary analysis because the data were collected prior to this revision, precluding prospective application of multiple imputation; however, the complete‐case sample preserved the internal consistency of the covariance structure required for valid regression estimates. While listwise deletion preserves internally consistent covariance structures, the substantial reduction in analytic sample size likely reduced statistical power to detect smaller effects and may limit generalizability. Moreover, listwise deletion assumes that data are missing completely at random (MCAR); if this assumption were not met, selection bias could have influenced estimates. For transparency, we report the model degrees of freedom (F [21, 225]), which reflect the complete‐case analytic sample size. Future studies incorporating planned multiple imputations and enhanced clinical measurement strategies may mitigate these limitations.

## 2 Results

### 2.1 Sample Characteristics

A total of 520 participants (mean age = 22.99, SD = 4.78 years, range = 18–59) completed the study. The majority were female (337 participants, 67.3%), Hispanic or Latino (262 participants, 52.1%), single (465 participants, 93.2%), and enrolled as full‐time students (468 participants, 90.0%). Participants were predominantly third‐year (128 participants, 25.7%) and fourth‐year (98 participants, 19.7%) undergraduates. Most reported a grade average of “B” (285 participants, 56.8%). Additional demographics and health risk factors are detailed in Table 1.

**Table 1:** Study demographics and health risk factors.

Variables	Frequency (%)
Age (Years) Valid *N* = 455	
18	48 (10.5)
19	51 (11.2)
20	48 (10.5)
21	53 (11.6)
≥ 22	255 (56.0)
Gender Valid *N* = 501	
Female	337 (67.3)
Male	164 (32.7)
Race/Ethnicity Valid *N* = 501	
Asian/Pacific Islander	138 (27.4)
Black	14 (2.8)
Hispanic/Latino	262 (52.1)
White	57 (11.3)
Other	32 (6.4)
Marital status Valid *N* = 499	
Married	34 (6.8)
Single	465 (93.2)
Employment status Valid *N* = 493	
Employed full‐time	37 (7.5)
Employed part‐time	238 (48.2)
Not employed	218(44.2)
Year in school Valid *N* = 498	
1st year undergraduate	81 (16.3)
2nd year undergraduate	64 (12.9)
3rd year undergraduate	128(25.7)
4th year undergraduate	98 (19.7)
5th year or more of undergraduate	73 (14.7)
Graduate or professional	52 (10.4)
Approximate grade average Valid *N* = 502	
A	138 (27.5)
B	285 (56.8)
C	69 (13.7)
Diagnosis of diabetes (valid *N* = 500)	12 (2.40)
Diagnosis of high blood pressure (valid *N* = 493)	25 (5.1)
Diagnosis of high cholesterol (valid *N* = 5495)	50 (10.1)
Having depression (PHQ‐9 scores ≥ 10) (valid *N* = 472)	116 (24.6)
Current smoker (valid *N* = 490)	21 (4.3)
Alcohol abstinent (valid *N* = 472)	295 (62.5)
Body mass index group Valid *N* = 368	
Under weight	32 (8.7)
Normal weight	181 (49.2)
Overweight	108 (29.3)
Obesity	47 (12.8)
Physical activity (valid *N* = 474)	158 (33.3)
Measured high blood pressure (valid *N* = 388)	27 (7.0)
Sleep duration Valid *N* = 498	
Sleep 6.0 h or less	231 (46.4)
Sleep 6.1–7.0 h	147 (29.5)
Sleep 7.1–8.0 h	89 (17.9)
More than 8.0 h	31 (6.2)

*Note:* Percentages are calculated based on valid cases (valid *N*) for each variable, excluding missing data.

### 2.2 General Health

For self‐rated general health, most participants rated their health as good (218 participants, 42%), followed by very good (151 participants, 29%), fair (68 participants, 13%), excellent (62 participants, 12%), and poor (16 participants, 3%). A *χ* ^2^ test of independence was employed between gender and self‐rated health, *χ* ^2^ (4) = 12.58, *p* < 0.05. In this study, male students reported significantly poorer self‐rated health compared to female students.

### 2.3 Health Behaviors

Health risk behaviors included current smoking (21 participants, 4.3%), any alcohol use in the past month (87 participants, 18.2%), and engagement in regular physical activity (158 participants, 33.3%). While the H‐SCALE assesses alcohol quantity and frequency, it categorizes participants based on adherence guidelines rather than isolating a specific “binge drinking” subgroup in the output. Most participants were abstinent from alcohol (295 participants, 62.5%) and nonsmokers who avoided secondhand smoke (385 participants, 74.1%). Regarding BMI categories, participants were primarily normal weight (181 participants, 49.2% among those reporting BMI), followed by overweight (108 participants, 29.3%), obese (47 participants, 12.8%), and underweight (32 participants, 8.7%). Female participants had significantly lower BMI (*M* = 23.72, SD = 5.49, *n* = 207) compared to male participants (*M* = 25.77, SD = 5.96, *n* = 116; t (222.38) = −3.06, *p *= 0.002). Weight management adherence was reported by 142 participants (30.1%). Adherence to a healthy diet was low (*n* = 37, 7.8%), while the majority of participants (*n* = 483, 92.2%) were classified as having an unhealthy diet based on DASH scores. Clinically measured high blood pressure was noted in a small subset (27 participants, 7.0% among those measured).

### 2.4 Sleep and Depression

Sleep duration varied, with approximately one‐third of participants reporting either 6 h (164 participants, 31.5%) or 7 h (153 participants, 29.4%) per night. About 93 participants (17.8%) reported an average of 8 h of sleep nightly. However, 62 participants (11.9%) reported sleeping about 5 h per night, and 17 participants (3.3%) reported sleeping 4 h or less per night. Analysis of PHQ‐9 data indicated that 116 participants (24.6%) met the criteria for depression.

### 2.5 Stress

Participant scores on the Perceived Stress Scale (PSS) ranged from 0 to 40, with a mean of 18.80 (SD = 6.19) and a median of 19. Female students reported significantly higher levels of perceived stress (*M* = 19.25, SD = 6.43) than their male counterparts (*M* = 17.88, SD = 4.56), *t* (357.747) = 2.411, *p* < 0.05. Using the mean (*M* = 18.80) of the PSS‐10 as the criterion for grouping, students with a perceived stress level **≥** 18.80 were considered to have a higher level of stress (*n* = 231, 48.7% of valid responses), and students with a perceived stress level < 18.80 were considered to have lower stress (*n* = 243, 51.3%).

### 2.6 Correlates of Demographics, Health Behaviors, Sleep, Depression With Stress Level

*χ* ^2^ tests indicated significant differences between participants with lower and higher stress levels (Table 2). Higher stress was significantly more common among third‐year undergraduate students (64 participants, 28.2%). Additionally, participants reporting ≤ 6 h of sleep per night (122 participants, 52.8%) and those classified as depressed (98 participants, 77.8%) had significantly higher stress levels.

**Table 2:** *χ* ^2^ Statistic of demographic and health risk factors by stress.

Variables	High stress *n* (%)	Low stress *n* (%)	*χ* ^2^	df	*p*	Cramér's *V*
Age (years)			4.12	4	0.390	—
18	23 (10.7)	24 (11.1)				
19	28 (13.1)	22 (10.1)				
20	25 (11.7)	18 (8.3)				
21	27 (12.6)	23 (10.6)				
≥ 22	111 (51.9)	130 (59.9)				
Gender			1.83	1	0.176	—
Female	158 (68.7)	149 (61.6)				
Male	72 (31.3)	93 (38.4)				
Year in school			11.27	3	0.010	0.166*
1st year	39 (17.2)	42 (19.4)				
2nd year	35 (15.4)	29 (13.4)				
3rd year	64 (28.2)	44 (20.4)				
4th year	36 (15.9)	62 (28.7)				
Depression (PHQ‐9 ≥ 10)			82.45	1	<0.001	0.498***
Yes	98 (77.8)	18 (10.6)				
No	28 (22.2)	152 (89.4)				
Sleep duration			12.84	3	0.005	0.167**
≤ 6.0 h	122 (52.8)	109 (45.0)				
6.1–7.0 h	58 (25.1)	89 (36.8)				
7.1–8.0 h	37 (16.0)	42 (17.4)				
> 8.0 h	14 (6.1)	17 (7.0)				
Physical activity			1.12	1	0.290	—
Adherent	68 (29.4)	90 (37.2)				
Not adherent	163 (70.6)	152 (62.8)				
BMI category			2.45	3	0.485	—
Underweight	11 (7.1)	21 (10.1)				
Normal weight	76 (49.0)	105 (50.5)				
Overweight	45 (29.0)	63 (30.3)				
Obesity	17 (11.0)	30 (14.4)				

*Note:* Percentages are calculated based on valid cases (valid *N*) for each variable, excluding missing data. High stress defined as PSS‐10 ≥ 18.80 (sample mean); low stress < 18.80. Fisher's exact test applied where expected cell counts < 5.

* *p *< 0.05

** *p *< 0.01

*** *p *< 0.001.

### 2.7 Stress Predictors

Multiple linear regression controlling for 21 predictors (demographics, health behaviors, clinical measures) yielded a significant model, *F*(21,225) = 7.426, *p*< 0.001, *R*² = 0.409. Four variables made statistically significant contributions: depressive symptoms (*β* = 0.509, *p*< 0.001), diastolic blood pressure (*β *= 0.153, *p*= 0.030), healthy diet adherence (*β *= −0.118, *p*= 0.040), and weight management (*β *= −0.156, *p*= 0.008). Higher depression and diastolic BP were associated with higher stress, while better dietary adherence and weight management were associated with lower stress. Systolic blood pressure, sleep duration, physical activity, smoking, alcohol, age, gender, race, marital status, BMI, self‐rated health, and cholesterol status were not significant predictors (Table 3).

**Table 3:** Predictors of stress among college students.

Predictor	*B*	SE	*β*	*t*	*p*	95% CI
(Constant)	22.28	4.75	—	4.69	< 0.001	[12.92, 31.63]
Age	0.09	0.07	0.067	1.20	0.230	[−0.06, 0.23]
Smoking	1.05	1.71	0.034	0.61	0.540	[−2.32, 4.42]
Self‐rated health (good)	−1.35	1.00	−0.076	−1.35	0.179	[−3.33, 0.63]
High cholesterol	1.35	1.05	0.068	1.28	0.202	[−0.73, 3.42]
Diabetes	2.14	2.51	0.050	0.85	0.394	[−2.80, 7.08]
GPA	−0.26	0.43	−0.032	−0.60	0.547	[−1.09, 0.58]
Physical activity	0.95	0.80	0.069	1.18	0.241	[−0.64, 2.53]
BMI	−0.10	0.07	−0.092	−1.39	0.165	[−0.23, 0.04]
Sleep duration (hours)	0.04	0.27	0.007	0.14	0.892	[−0.49, 0.56]
**PHQ‐9 depression score**	**0.51**	**0.06**	**0.509**	**8.21**	**< 0.001** *	**[0.39, 0.64]**
Mean systolic BP	−0.06	0.03	−0.136	−1.87	0.063	[−0.13, 0.00]
**Mean diastolic BP**	**0.11**	**0.05**	**0.153**	**2.19**	**0.030** *	**[0.01, 0.20]**
White (vs. other race)	−0.05	1.63	−0.003	−0.03	0.975	[−3.27, 3.17]
**DASH diet adherence**	−**1.18**	**0.57**	−**0.119**	−**2.07**	**0.040** *	**[−2.31, −0.05]**
**Weight management score**	−**0.11**	**0.04**	−**0.156**	−**2.67**	**0.008** *	**[−0.19, −0.03]**
Hispanic (vs. non‐Hispanic)	−0.58	1.31	−0.048	−0.45	0.656	[−3.16, 2.00]
Asian American	1.63	1.41	0.118	1.16	0.248	[−1.15, 4.41]
Depression prevalence (PHQ ≥ 10)	0.34	1.05	0.019	0.32	0.751	[−1.74, 2.41]
Not married	−0.16	1.50	−0.006	−0.10	0.918	[−3.10, 2.79]
Alcoholic drinks per week	0.02	0.05	0.026	0.39	0.695	[−0.07, 0.11]
Alcohol abstinent	−0.80	0.76	−0.065	−1.05	0.293	[−2.31, 0.70]

*Note:* Bold indicates statistically significant predictors.

Sample: *n* = 247 (complete cases after listwise deletion).

Model statistics: F(21, 225) = 7.426, *p* < 0.001, *R*² = 0.409, Adjusted *R*² = 0.354.

*p < 0.05.

## 3 Discussion

This study aimed to identify specific modifiable health behaviors associated with perceived stress among undergraduate college students. Given the cross‐sectional design, identified associations represent statistical relationships rather than causal pathways. Participants demonstrated relatively healthier behaviors compared to national averages. The study participants had a lower smoking rate (4.18% vs. 17.6%), obesity rate (12.8% vs. 19.5%), physical activity rate (33.3% vs. 38%), and alcohol intake (18.2% vs. 51.5%) compared to national survey data for young adults aged 18–24. These differences may be partially attributed to methodological variations, such as differing measures and reference periods. For example, our smoking measure assessed cigarette or cigar usage over the past week, whereas national surveys typically inquire about usage within the past month and often distinguish vaping separately. Currently, e‐cigarettes (vaping) have become the predominant tobacco product among the youth. From 2019 to 2023, e‐cigarette use among college students increased, with rates of use in the past 30 days rising from 14.3% to 15.9% [10]. Given current trends, prevention strategies and access to cessation support for college students are essential.

The National Sleep Foundation [38] recommends an average of 7–9 h of sleep for young adults aged 18–25. Insufficient sleep is a well‐established risk factor associated with higher stress levels, poor general health, and increased substance use [3, 39]. Promoting healthy sleep hygiene through consistent sleep schedules, improved sleep environments, and education about sleep‐related health outcomes could substantially enhance college students' well‐being.

Overall, our findings regarding average stress levels align with previous research suggesting moderate stress among college students [1, 40, 41]. However, we noted significant variability within our sample. Notably, third‐year students exhibited higher stress levels. This aligns with previous literature indicating that stress, anxiety, and depression peak during students' first three years of college and often decline by graduation [42, 43, 44].

Our analysis also identified higher stress among female students compared to their male counterparts, consistent with previous national findings [1]. Young females aged 18–34 frequently report feeling overwhelmed by stress at notably higher rates compared to older adults and males [1]. Additionally, we observed a strong association between elevated depressive symptoms and increased stress, corroborating previous studies [24, 45]. The strong association between depression and stress likely reflects substantial shared variance in negative affect and overlapping symptomatology, which may have attenuated associations between stress and other predictors in the multivariable model. A recent survey found that one in five young women and one in ten young men aged 25 or younger experience major depression [45]. These findings underscore the need for enhanced mental health support and resources for young adults to promote well‐being.

The significant association between diastolic blood pressure and stress, rather than systolic pressure, may reflect the autonomic nervous system's response to stress in young adults. Stress activates the sympathetic nervous system, triggering hormonal cascades that increase peripheral vascular resistance and elevate blood pressure, with diastolic pressure particularly sensitive to stress‐induced vasoconstriction in young adults [46, 47].

Interventions addressing stress are critical for academic and personal success among college students [24, 48]. Our study identified associations between depression, dietary habits, weight management, and elevated diastolic blood pressure with perceived stress. Adherence to healthy diets and weight management practices was associated with lower stress, underscoring their potential importance for well‐being. These associations suggest targeted lifestyle interventions may benefit stress management among college populations. Given that high stress frequently manifests physically through symptoms such as headaches, fatigue, and depression [1], promoting healthy behaviors might mitigate these adverse outcomes.

Our findings align with prior research linking higher stress with depression [1, 24, 48, 49] and elevated blood pressure [46, 50], emphasizing the importance of addressing these interconnected health issues. A recent study of community college students used a randomized controlled trial and concluded that higher stress was associated with a higher prevalence of obesity, skipping meals, and smoking [12]. A study of 14,870 college students found that increased stress was associated with poorer sleep quality, higher alcohol and tobacco use, and lower overall general health [3]. These findings highlight the interdependent relationship between physical and mental health and support a comprehensive approach to health promotion aimed at optimizing overall student well‐being.

This study provides an essential baseline for modifiable risk behaviors and stress in a diverse college student sample. Our findings can inform tailored interventions that incorporate both primary prevention (promoting healthy behaviors) and secondary prevention (managing stress and related conditions). Public health professionals and university health programs may utilize this data to create evidence‐based, targeted health promotion strategies.

Our findings revealed gender‐specific differences in sleep duration and stress. Females typically reported less sleep and higher stress levels than males. Thus, gender‐sensitive approaches in health counseling and campus wellness programs are recommended. Encouraging better sleep practices, especially among female students, may effectively lower their perceived stress.

## 4 Limitations

Several limitations should be noted. First, self‐report measures inherently introduce response bias and accuracy concerns. Specifically, assessments of health conditions, stress, sleep, and lifestyle behaviors were self‐reported. Second, the cross‐sectional design precludes causal inference. Third, the lack of data on students' academic majors limits insights into academic‐specific stressors. Fourth, we did not distinguish between traditional cigarette smoking and vaping, despite their distinct health risks. Future studies should differentiate these behaviors. Fifth, sleep was measured solely by duration, neglecting qualitative aspects such as disturbances and overall sleep quality; future studies should include comprehensive sleep assessments. Sixth, the incentive provided for participation ($5 gift card) may have introduced selection bias. Finally, the convenience sampling of predominantly female and Hispanic students restricts generalizability beyond similar populations.

We also did not measure pandemic‐specific stressors or account for time‐based differences in data collection during the COVID‐19 pandemic, which may have significantly impacted stress levels. This omission represents a potential confounder. Moreover, our analysis examined only main effects, and we did not explore interactions (e.g., sleep × depression, gender × depression) or potential mediating variables. Although interaction and mediation analyses can be informative, we did not estimate these effects for three reasons. First, the cross‐sectional design precludes establishing the temporal ordering required for causal mediation, limiting the interpretability of any indirect pathways. Second, listwise deletion yielded a reduced analytic sample (*n *= 247) relative to the number of predictors, constraining statistical power and model stability for detecting typically small higher‐order effects, and increasing the risk of both Type I error inflation and unstable estimates in the absence of strong a priori hypotheses and appropriate multiplicity control. Third, several measures were brief (e.g., sleep duration assessed by a single item; brief adherence indices), which may attenuate sensitivity to detect subtle interactions or indirect effects. In line with our prespecified aims, we therefore emphasized main‐effect associations to preserve interpretability and analytic rigor. Future longitudinal studies with larger effective samples, multi‐item constructs, and principled missing‐data approaches (e.g., multiple imputation) are recommended to test interaction and mediation effects robustly. Additionally, dichotomizing stress at the sample mean for descriptive analyses (Table 2) may have reduced statistical efficiency, though primary regression analyses appropriately used continuous stress scores. Future research exploring these dimensions could further clarify the mechanisms underlying stress among college students.

## 5 Conclusion

This study identified important associations between modifiable lifestyle behaviors (healthy diet and weight management), depression, diastolic blood pressure, and stress among college students. Elevated depressive symptoms and diastolic blood pressure were associated with higher stress, while healthy dietary practices and weight management behaviors were associated with lower stress. These findings suggest that depressive symptoms, diastolic blood pressure, and specific self‐care behaviors co‐occur with perceived stress in this university sample; longitudinal studies are needed to clarify temporal ordering.

### 5.1 Relevance for Clinical Practice

Young adults frequently establish lifelong health habits during college. These associations suggest that comprehensive campus health programs that address dietary habits, weight management, and mental health support may benefit student well‐being, though longitudinal and intervention studies are needed to establish temporal relationships and the efficacy of interventions. Policymakers, clinicians, and public health professionals should consider these modifiable factors when designing campus wellness programs.

## Author Contributions

**Ayman Tailakh:** conceptualization, data curation, investigation, methodology, project administration, resources, supervision, visualization, writing – original draft, writing – review and editing. **Ali Ahmad Ammouri:** formal analysis, investigation, methodology, validation, writing – original draft, writing – review and editing.

## Funding

The authors have nothing to report.

## Conflicts of Interest

The authors declare no conflicts of interest.

## Transparency Statement

The corresponding author, Ayman Tailakh, affirms that this manuscript is an honest, accurate, and transparent account of the study being reported; that no important aspects of the study have been omitted; and that any discrepancies from the study as planned (and, if relevant, registered) have been explained.

## Data Availability

The data that support the findings of this study are available from the corresponding author upon reasonable request.
